# Supplementary material for: Development and Application of an Automated Raman Sensor for Bioprocess Monitoring: From the Laboratory to an Algae Production Platform
Source: Sensors (Basel). 2023 Dec 11;23(24):9746. doi: 10.3390/s23249746 (PMC10747176; doi:10.3390/s23249746)
Supplement: Supplementary file 1 [file sensors-23-09746-s001.zip › sensors-2700853-supplementary.pdf]

Supplementary Materials

Table S1. Overview of the main Raman bands observed during the analysis of *Parachlorella Kessleri.* Abbreviations indicate: ν, stretching; δ, bending; ρ, rocking s, symmetrical; as, asymmetrical.

RAMAN SHIFT (cm^{-1})	ASSIGNMENT OF BANDS	MOLECULES OF INTEREST
479	δ (C-C-C)	Carbohydrates
744	ν (H-C-O) / δ (N-C-C)	Carbohydrates / chlorophyll *a*
865	C_4N^+, ν_s (O-C-C-N)	Phospholipids
915	δ (N-C-C), δ (C-C-C)	Chlorophyll a
988	δ ($C-H_3$)	Chlorophyll a
997	ν (C-C)	Beta-carotene
1009	ρ (C-C)	Carotenoids
1157	ν (C-C)	Carotenoids
1191	δ (C-H)	Beta-carotene
1444	δ ($C-H_2$)	Lipids
1524	ν (C=C)	Carotenoids
1600–1700	Amide I	Proteins
1660	ν $(C=C)_{cis}$	Lipids
1750	ν (C=O)	Lipids
2850	ν_s ($C-H_2$)	Lipids
2885	ν_s ($C-H_3$)	Lipids
2940	ν_{as} ($C-H_2$)	Lipids
2970	ν_{as} ($C-H_3$)	Lipids
3008	ν_{as} (=C-H)	Lipids

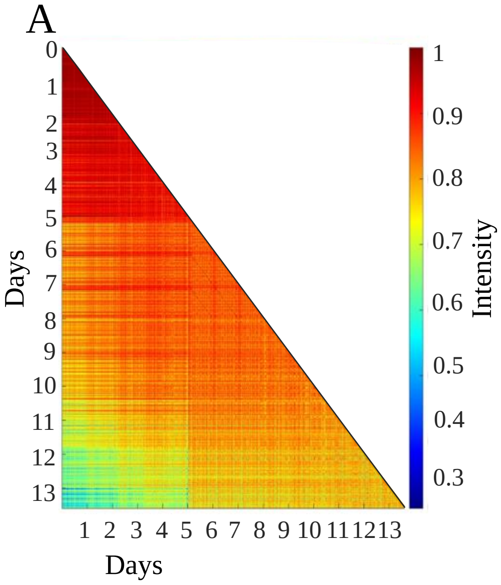

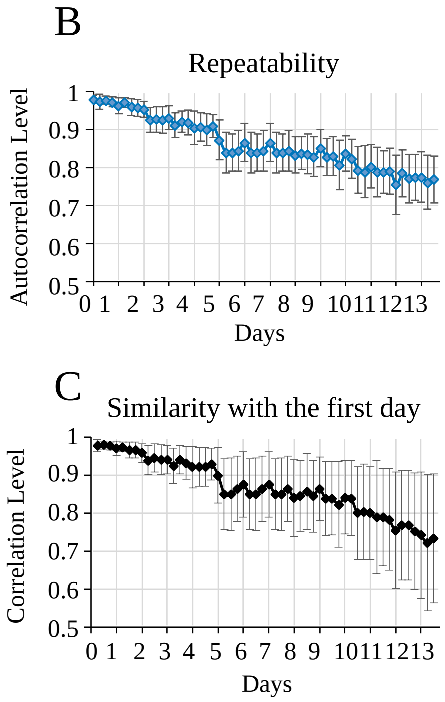

	Mean value	Standard deviation
Total correlation	0.82	0.09
Day 00 to 04	0.93	0.04
Day 05 to 10	0.83	0.07
Day 10 to 13	0.78	0.07

Figure S1. A. 2D correlation map of all spectra, with a correlation table covering the 14 days of culture in a 100-L tubular airlift bioreactor. The colour of each map point represents the level of correlation between two spectra, from red (highest correlation) to blue (lowest correlation). B. Repeatability of spectra measured by the autocorrelation level between 50 spectra recorded in the same time window. C. Evolution of the correlation level of the spectra compared by correlation with the first day

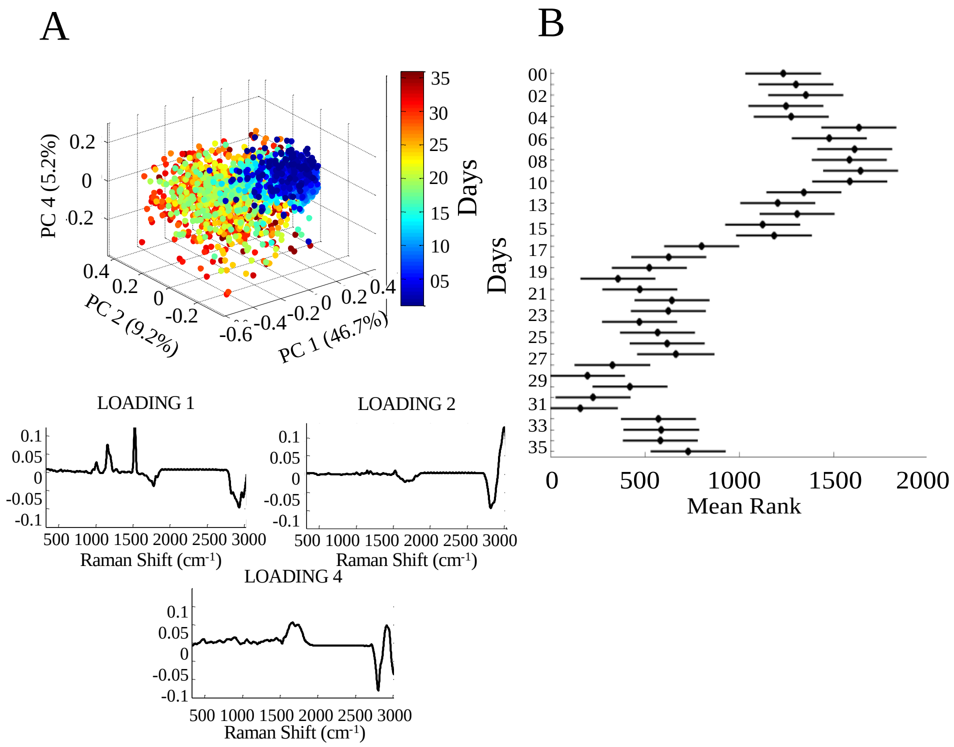

Figure S2. Three-dimensional representation of the principal component analysis (PCA) of 36 days of culture in a 1-L laboratory-scale photobioreactor (PC1 46.7%; PC2 9.2%; PC4 5.2%) and their three respective loadings. B. Kruskal-Wallis one-way ANOVA test, based on PCA loading 1, representing the variance of the spectra over the 36 days in a 1-L laboratory-scale culture.

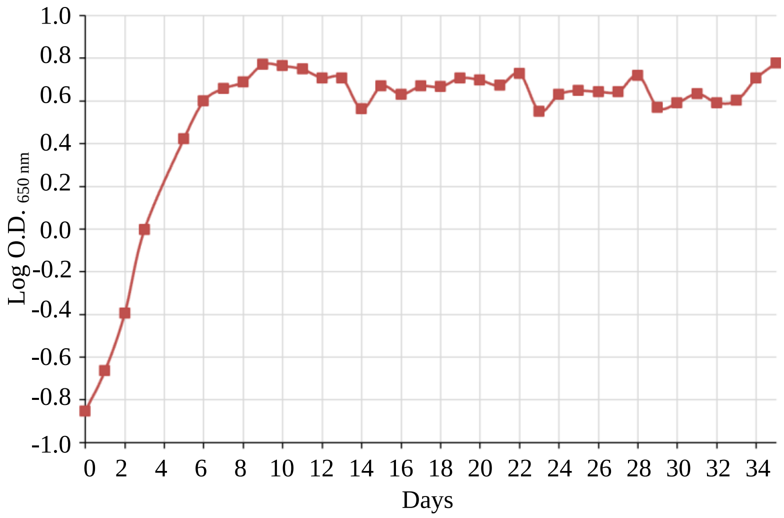

Figure S3. Monitoring of *Parachlorella kessleri* in Bold Basal Medium in a 1-L tubular airlift photobioreactor from day 0 to day 35. Cell density increase during cell growth until nitrogen limitation (tenth day), stabilizing afterwards.

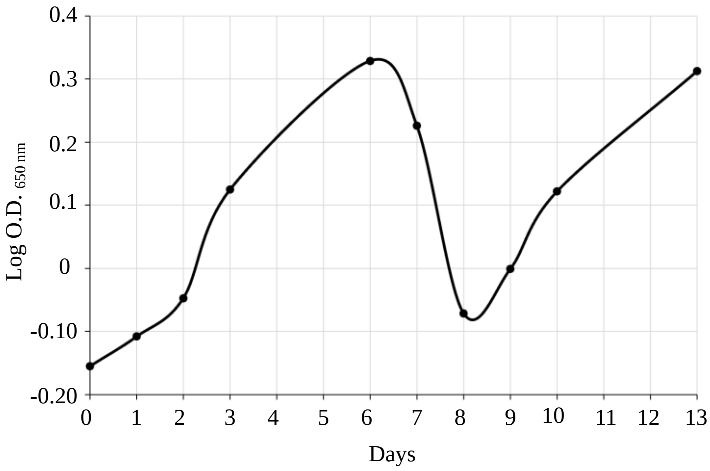

Figure S4. Monitoring of *Parachlorella kessleri* in Bold Basal Medium in a 100-L tubular airlift photobioreactor from day 0 to day 13. Cell density increase during cell growth until 6th when another 50L of BBM medium was added, the cell density increased again after the 8th day
